# Crystal structure of bis­[4-(1*H*-pyrrol-1-yl)phen­yl] ferrocene-1,1′-di­carboxyl­ate: a potential chemotherapeutic drug

**DOI:** 10.1107/S2056989015007446

**Published:** 2015-04-22

**Authors:** Wanda I. Pérez, Arnold L. Rheingold, Enrique Meléndez

**Affiliations:** a University of Puerto Rico, Department of Chemistry, PO Box 9019, Mayaguez, Puerto Rico 00681, USA; b University of California-San Diego, Department of Chemistry, Urey Hall 5128, 9500 Gilman Drive, La Jolla, CA 92093-0358, USA

**Keywords:** crystal structure, disubstituted ferrocene, anti­proliferative, chemotherapeutic drug, MCF-7, pyrrole

## Abstract

The title iron(II) complex, [Fe(C_16_H_12_NO_2_)_2_], crystallizes in the ortho­rhom­bic space group *Pbca* with the Fe^2+^ cation positioned on an inversion center. The cyclo­penta­dienyl (Cp) rings adopt an *anti* conformation in contrast with other substituted ferrocenes in which the Cp rings appear in a nearly eclipsed conformation. The Cp and the aromatic rings are positioned out of the plane, with a twist angle of 70.20 (12)°, and the C(Cp)—C(CO) bond length is shorter than a typical C—C single bond, which suggests a partial double-bond character and delocalization with the Cp π system. The structure of the complex is compared to other functionalized ferrocenes synthesized in our laboratory.

## Abstract 2

The solid-state structure of a disubstituted ferrocene with electrochemically active pendant groups has been determined. This is a potential chemotherapeutic drug.

## Chemical context   

The gold standard of treatment for breast cancer has traditionally been cisplatin, a metal-based agent. Its administration, alone or in combination with other drugs, is also highly effective against various other types of cancers, including ovarian, head and neck, bladder, testicular and lung cancers (Galanski *et al.*, 2005 ▸; Sandler *et al.*, 2011 ▸). However, its clinical use suffers from major drawbacks, such as severe toxic side effects including neurotoxicity, hepatotoxicity, and nephrotoxicity (Pabla & Dong, 2008 ▸), as well as a drug-resistance phenomenon which leads to unsuccessful treatment (Dempke *et al.*, 2000 ▸). Consequently, other metal-based drugs have been investigated, among them ferrocenes (Köpf-Maier *et al.*, 1984 ▸). Ferrocene has the versatility of easy functionalization providing a fertile field for structural modification and to study structure–activity relationship (SAR).

Our group has been working in this field for many years, leading to exciting and biologically active ferrocenes. A wide variety of pendant (functional) groups have been attached or linked to the Cp ring to tailor the anti-proliferative properties of ferrocene, many of them with great success (Braga & Silva, 2013 ▸; Gasser *et al.*, 2011 ▸; Jaouen & Metzler-Nolte, 2010 ▸; Fouda *et al.*, 2007 ▸; Jaouen, 2006 ▸; van Staveren & Metzler-Nolte, 2004 ▸; Nguyen *et al.*, 2009 ▸; Top *et al.*, 2003 ▸; Vessières *et al.*, 2005 ▸, 2006 ▸; Meléndez, 2012 ▸; Vera *et al.*, 2011 ▸, 2014 ▸). Lately, a new range of organic chemotherapeutic compounds have been studied using pyrrole derivatives. These pyrrole derivatives have revealed good anti-proliferative activity and an increase in membrane permeability, allowing the compounds to reach the nucleus (Ghorab *et al.*, 2014 ▸; Abou El Ella *et al.*, 2008 ▸; Chatzopoulou *et al.*, 2014 ▸; Mohamed *et al.*, 2013 ▸; Hassan *et al.*, 2009 ▸; Esteves *et al.*, 2010 ▸; Clark *et al.*, 2007 ▸; Merighi *et al.*, 2003 ▸). Therefore, we functionalized ferrocene with a pyrrole, 4-(1*H*-pyrrol-1-yl)phenol, obtaining three new ferrocenes: 1,1′-4-(1*H*-pyrrol-1-yl)phenyl ferrocenedi­carboxyl­ate, 1,4-(1*H*-pyrrol-1-yl)phenyl, 1′-carboxyl ferrocene­carboxyl­ate (Fc-(CO_2_-Ph-4-Py)CO_2_H) and 4-(1*H*-pyrrol-1-yl)phenyl ferro­cene­acetyl­ate (Fc-CH_2_CO_2_-Ph-4-Py). We investigated their biological activities on breast cancer cell line (MCF-7) and among these ferrocenes, 1,1′-4-(1*H*-pyrrol-1-yl)phenyl ferrocenedi­carboxyl­ate (I) was shown to be most active in this series (Pérez *et al.*, 2015 ▸). Nevertheless, the solid-state structure of (I) has been elusive (Pérez *et al.*, 2015 ▸). The importance of this complex is the incorporation of pyrrole groups, which are derivatives of biologically active compounds, as well as pyrrole being an electrochemically active group precursor of polymeric mat­erial. In addition, ferrocene anti­cancer activity has been associated with its redox behavior and the capability to produce reactive oxygen species (ROS) (Acevedo *et al.*, 2012 ▸; Kovjazin *et al.*, 2003 ▸; Tabbi *et al.*, 2002 ▸; Osella *et al.*, 2005 ▸). Thus, the attachment of an electrochemically active group on ferrocene could potentiate the production of ROS and enhance its anti­cancer activity.
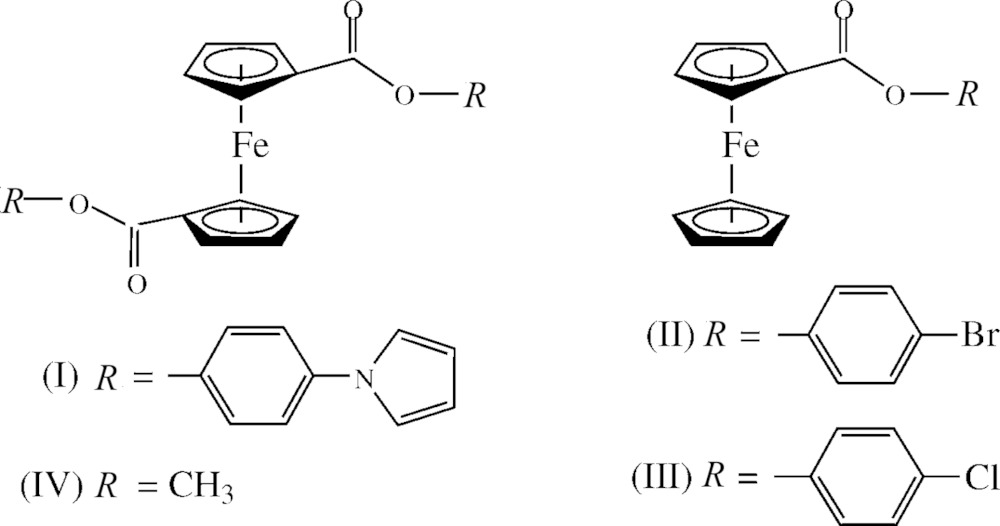


Given that the solid-state structure of this complex is not available, we determined the crystal structure of bis­[4-(1*H*-pyrrol-1-yl)phen­yl] ferrocene-1,1′-di­carboxyl­ate, (I). Additionally, we compared the obtained crystal structure with other functionalized ferrocenes synthesized in our laboratory *viz.*: 4-bromo­phenyl (II) and 4-chloro­phenyl ferrocene­carboxyl­ate (III) (Vera *et al.*, 2014 ▸), and 1,1′-methyl ferrocenedi­carboxyl­ate (IV) (Gao *et al.*, 2009 ▸).

## Structural commentary   

**Figure 1:**
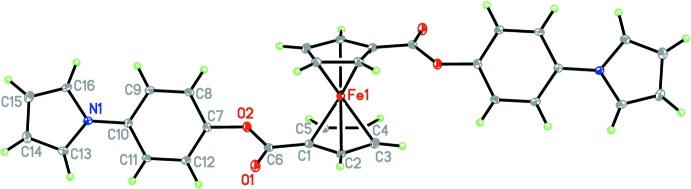
The mol­ecular structure of (I), with displacement ellipsoids drawn at the 30% probability level. Unlabelled atoms are related to labelled ones by the symmetry operation −*x*, −*y*, −*z*.

The asymmetric unit contains one half-mol­ecule since Fe^2+^ lies on an inversion center, Fig. 1 ▸. This symmetry is implied by the NMR data where only one set of signals were found for H2/H5 and H3/H4 of the Cp rings, as well as the H2/H6 and H3/H5 of the phenyl and H2/H5 and H3/H4 of the pyrrole groups. Consequently, the Cp rings adopt a perfect *anti* conformation. The average Fe—C(Cp) bond length is 2.044 (10) Å, which is very similar to that reported for ferrocene (Dunitz *et al.*, 1956 ▸) and other structures previously reported by our lab (Vera *et al.*, 2014 ▸; Gao *et al.*, 2009 ▸). The Fe—C bond length of the substituted carbon [Fe—C1 2.032 (2) Å] is shorter that the remaining Fe—C bond lengths due to the inductive effect of the carboxyl­ate on the Cp ring. The twist angles between the Cp ring and the carboxyl­ate and the Cp ring and the aromatic ring are 14.4 (3)° (above the Cp plane) and 70.20 (12)°, respectively.

To put it in perspective, we compare (I) with previously synthesized ferrocenes in our group containing only one Cp functionalized and a phenyl group attached to the carboxyl­ate, but with Br and Cl instead of pyrrole in the 4-position, (II) and (III) (CCDC 949002 and 949003, Vera *et al.*, 2014 ▸). First, in the 4-bromo­phenyl and 4-chloro­phenyl derivatives, the Cp rings are positioned in a nearly eclipsed conformation and parallel with stagger angles < 3° and Cp tilt angles of 0.48–1.25°. In contrast, (I) has a perfect *anti* conformation. The carbonyl carbon of (I) has a distorted trigonal–planar geometry, analogous to the 4-chloro­phenyl and 4-bromo­phenyl ferrocene­carboxyl­ates. The twist angles between the Cp ring and the carboxyl­ate for 4-bromo and 4-chloro­phenyl ferrocene­carboxyl­ates (6.75–10.15°) are smaller than that of the subject complex, 14.4 (3)°. Additionally, as mentioned previously, the carbonyl oxygen of (I) lies above the Cp plane whereas for the bromo and chloro derivatives, the carbonyl oxygens lie below the Cp plane. The twist angle between the Cp and the aromatic ring is 70.20 (12)° in (I), while in (II) and (III) the two rings are positioned at higher angles, approaching a perpendicular position.

The average Fe—C(Cp*) bond lengths of the substituted Cp rings in the 4-bromo and 4-chloro­phenyl derivatives are identical, within experimental error, as in (I) [2.044 (13) Å]. As mentioned before, the Fe—C bond length where the pendant group is attached is substanti­ally shorter than the remaining Fe—C(Cp) distances. The same bonding pattern is also observed for the 4-bromo and 4-chloro­phenyl ferrocene­carboxyl­ates. The C(Cp)—C(CO) bond length in (I), C1—C6, is shorter than a typical C—C single bond, [1.473 (3) versus 1.54 Å (single bond); Pauling, 1960 ▸]. This suggests partial double-bond character and delocalization with the Cp π system in analogous manner to that for the 4-bromo and 4-chloro derivatives.

**Figure 2:**
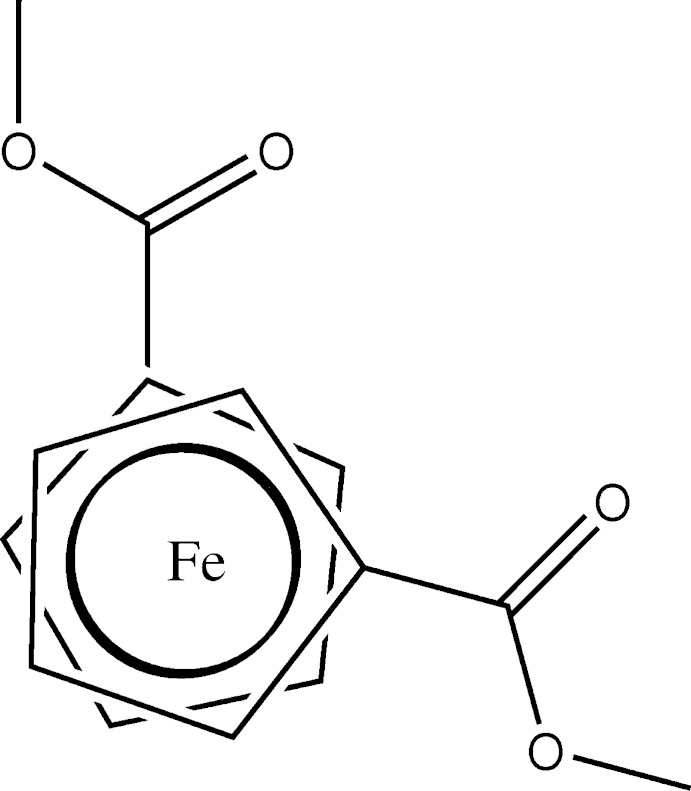
A Newman projection of Fe(C_5_H_4_CO_2_CH_3_)_2_.

In the structure of the disubstituted ferrocene Fe(C_5_H_4_CO_2_CH_3_)_2_, (IV) (Gao *et al.*, 2009 ▸), the average Fe—C(Cp) bond lengths are 2.048 (11)/2.049 (14) Å, similar to the title complex but the Cp rings adopt almost an eclipsed conformation with a stagger angle of 2.37° (Fig. 2 ▸). In addition, the functional groups are not positioned perfectly *anti* to each other. The Fe—C(Cp)—C(CO) bond in (IV) [1.477 (4) Å] is notably shorter than a typical C—C single bond (1.54 Å), in a similar manner to the title complex, suggesting delocalization with the Cp π system.

Finally, (I) contains two π ring systems, 4-(1*H*-pyrrol-1-yl)phenyl, which in principle could be involved in intra­molecular π–π or C—H⋯π stacking similar to other 1,1′-disubstituted ferrocenes with an extended π ring system (Okabe *et al.*, 2009 ▸; Togni *et al.*, 1994 ▸; Gelin & Thummel, 1992 ▸). However, such π–π or C—H⋯π stacking is not observed in (I) since the Cp rings adopt an *anti* conformation.

## Synthesis and crystallization   

The synthesis of (I) was accomplished by treating 1,1′-ferrocenedi­carb­oxy­lic acid with oxalyl chloride according to our recently published procedure (Pérez *et al.*, 2015 ▸). ^1^H NMR (500 MHz, CDCl_3_) (δ p.p.m.): 7.37 (2H, *d*, ph; ^3^ *J* = 8.8 Hz), 7.25 (2H, *d*, py; ^3^ *J* = 2.8 Hz), 7.03 (2H, *dd*, ph; ^3^ *J* = 1.3 Hz), 6.34 (2H, *dd*, py; ^3^ *J* = 1.6 Hz), 5.08 (2H, overlapping doublets, AA′, Cp), 4.64 (2H, overlapping doublets, BB′, Cp). ^13^CNMR (125 MHz, CDCl_3_) (δ p.p.m.): 169.0 (C=O), 148.3, 138.6, 122.9, 121.5, 119.5, 110.5, 73.4, 72.4, 72.0. Analysis calculated for C_32_H_24_O_4_FeN_2_: C, 69.05; H, 4.40; found: C, 68.62; H, 4.46.

Crystallization of (I) was performed inside an NMR tube containing CD_2_Cl_2_ for a period of two weeks, obtaining block-shaped orange crystals suitable for X-ray diffraction.

## Refinement   

Crystal data, data collection and structure refinement details are summarized in Table 1 ▸. H atoms were positioned in idealized locations (C_(6)_—H = 0.95, C_(5)_—H = 1.00 Å with *U*_iso_(H) = 1.2*U*_eq_(C).

## Supplementary Material

Crystal structure: contains datablock(s) I. DOI: 10.1107/S2056989015007446/bg2552sup1.cif

Structure factors: contains datablock(s) I. DOI: 10.1107/S2056989015007446/bg2552Isup2.hkl

CCDC reference: 1054149

Additional supporting information:  crystallographic information; 3D view; checkCIF report

## Figures and Tables

**Table 1:** Experimental details

Crystal data	
Chemical formula	[Fe(C_16_H_12_NO_2_)_2_]
*M*_r_	556.38
Crystal system, space group	Orthorhombic, *P* *b* *c* *a*
Temperature (K)	100
*a*, *b*, *c* ()	10.6386(15), 7.3948(10), 30.554(4)
*V* (^3^)	2403.7(6)
*Z*	4
Radiation type	Mo *K*
(mm^1^)	0.67
Crystal size (mm)	0.28 0.26 0.23

Data collection	
Diffractometer	Bruker APEXII CCD
Absorption correction	Multi-scan (*SADABS*; Bruker, 2010 ▸)
*T*_min_, *T*_max_	0.833, 0.877
No. of measured, independent and observed [*I* > 2(*I*)] reflections	12444, 2999, 2247
*R*_int_	0.077
(sin /)_max_ (^1^)	0.669

Refinement	
*R*[*F* ^2^ > 2(*F* ^2^)], *wR*(*F* ^2^), *S*	0.044, 0.117, 1.02
No. of reflections	2999
No. of parameters	178
H-atom treatment	H-atom parameters constrained
_max_, _min_ (e ^3^)	0.34, 0.62

Computer programs: *APEX2* and *SAINT* (Bruker, 2010 ▸), *SHELXS97* and *SHELXTL* (Sheldrick, 2008 ▸) and *SHELXL2013* (Sheldrick, 2015 ▸).

## supplementary crystallographic information

### Crystal data

**Table:**

[Fe(C_16_H_12_NO_2_)_2_]	*D*_x_ = 1.537 Mg m^−^^3^
*M**_r_* = 556.38	Mo *K*α radiation, λ = 0.71073 Å
Orthorhombic, *P**b**c**a*	Cell parameters from 2807 reflections
*a* = 10.6386 (15) Å	θ = 2.7–28.1°
*b* = 7.3948 (10) Å	µ = 0.67 mm^−^^1^
*c* = 30.554 (4) Å	*T* = 100 K
*V* = 2403.7 (6) Å^3^	Block, orange
*Z* = 4	0.28 × 0.26 × 0.23 mm
*F*(000) = 1152	

### Data collection

**Table:**

Bruker APEXII CCD diffractometer	2247 reflections with *I* > 2σ(*I*)
φ and ω scans	*R*_int_ = 0.077
Absorption correction: multi-scan (*SADABS*; Bruker, 2010)	θ_max_ = 28.4°, θ_min_ = 2.7°
*T*_min_ = 0.833, *T*_max_ = 0.877	*h* = −13→14
12444 measured reflections	*k* = −9→9
2999 independent reflections	*l* = −37→40

### Refinement

**Table:**

Refinement on *F*^2^	0 restraints
Least-squares matrix: full	Hydrogen site location: inferred from neighbouring sites
*R*[*F*^2^ > 2σ(*F*^2^)] = 0.044	H-atom parameters constrained
*wR*(*F*^2^) = 0.117	*w* = 1/[σ^2^(*F*_o_^2^) + (0.047*P*)^2^ + 0.928*P*] where *P* = (*F*_o_^2^ + 2*F*_c_^2^)/3
*S* = 1.01	(Δ/σ)_max_ < 0.001
2999 reflections	Δρ_max_ = 0.34 e Å^−^^3^
178 parameters	Δρ_min_ = −0.62 e Å^−^^3^

### Special details

**Table:**

Geometry. All e.s.d.'s (except the e.s.d. in the dihedral angle between two l.s. planes) are estimated using the full covariance matrix. The cell e.s.d.'s are taken into account individually in the estimation of e.s.d.'s in distances, angles and torsion angles; correlations between e.s.d.'s in cell parameters are only used when they are defined by crystal symmetry. An approximate (isotropic) treatment of cell e.s.d.'s is used for estimating e.s.d.'s involving l.s. planes.

### Fractional atomic coordinates and isotropic or equivalent isotropic displacement parameters (Å^2^)

**Table:**

	*x*	*y*	*z*	*U*_iso_*/*U*_eq_	
Fe1	0.5000	0.5000	0.5000	0.01323 (14)	
O1	0.23108 (15)	0.4748 (2)	0.41347 (5)	0.0213 (4)	
O2	0.41465 (13)	0.3430 (2)	0.39352 (4)	0.0173 (3)	
N1	0.37269 (16)	0.3889 (2)	0.21120 (5)	0.0133 (4)	
C1	0.3680 (2)	0.3523 (3)	0.46783 (7)	0.0149 (4)	
C2	0.3145 (2)	0.4294 (3)	0.50667 (6)	0.0169 (4)	
H2A	0.2415	0.5142	0.5081	0.020*	
C3	0.3857 (2)	0.3643 (3)	0.54286 (7)	0.0216 (5)	
H3A	0.3716	0.3970	0.5742	0.026*	
C4	0.4812 (2)	0.2469 (3)	0.52692 (7)	0.0191 (5)	
H4A	0.5454	0.1827	0.5451	0.023*	
C5	0.4712 (2)	0.2403 (3)	0.48035 (7)	0.0166 (4)	
H5A	0.5262	0.1693	0.4601	0.020*	
C6	0.3264 (2)	0.3975 (3)	0.42318 (6)	0.0144 (4)	
C7	0.3923 (2)	0.3684 (3)	0.34853 (6)	0.0145 (4)	
C8	0.48316 (19)	0.4601 (3)	0.32540 (7)	0.0155 (4)	
H8A	0.5502	0.5182	0.3404	0.019*	
C9	0.47630 (19)	0.4673 (3)	0.27993 (7)	0.0151 (4)	
H9A	0.5398	0.5288	0.2639	0.018*	
C10	0.37736 (19)	0.3853 (2)	0.25769 (6)	0.0121 (4)	
C11	0.28353 (19)	0.2994 (3)	0.28206 (7)	0.0149 (4)	
H11A	0.2137	0.2469	0.2674	0.018*	
C12	0.29095 (19)	0.2899 (3)	0.32731 (6)	0.0154 (4)	
H12A	0.2272	0.2302	0.3436	0.018*	
C13	0.2795 (2)	0.3154 (3)	0.18518 (7)	0.0174 (4)	
H13A	0.2077	0.2518	0.1954	0.021*	
C14	0.3076 (2)	0.3494 (3)	0.14240 (7)	0.0195 (5)	
H14A	0.2587	0.3153	0.1177	0.023*	
C15	0.4232 (2)	0.4449 (3)	0.14143 (7)	0.0213 (5)	
H15A	0.4661	0.4862	0.1161	0.026*	
C16	0.4613 (2)	0.4663 (3)	0.18374 (7)	0.0184 (4)	
H16A	0.5364	0.5248	0.1929	0.022*	

### Atomic displacement parameters (Å^2^)

**Table:**

	*U*^11^	*U*^22^	*U*^33^	*U*^12^	*U*^13^	*U*^23^
Fe1	0.0159 (2)	0.0111 (2)	0.0127 (2)	−0.00228 (16)	−0.00142 (16)	0.00108 (15)
O1	0.0196 (9)	0.0274 (8)	0.0169 (8)	0.0076 (7)	−0.0004 (6)	−0.0006 (6)
O2	0.0162 (8)	0.0220 (8)	0.0137 (7)	0.0029 (6)	−0.0003 (6)	−0.0013 (6)
N1	0.0123 (8)	0.0123 (8)	0.0154 (8)	−0.0003 (7)	0.0015 (7)	−0.0003 (6)
C1	0.0164 (10)	0.0125 (9)	0.0160 (10)	−0.0039 (8)	0.0003 (8)	−0.0003 (7)
C2	0.0161 (10)	0.0168 (10)	0.0179 (10)	−0.0040 (9)	0.0031 (8)	0.0006 (8)
C3	0.0292 (12)	0.0190 (11)	0.0165 (11)	−0.0081 (10)	0.0015 (9)	0.0016 (8)
C4	0.0232 (12)	0.0139 (10)	0.0201 (11)	−0.0055 (9)	−0.0051 (9)	0.0046 (8)
C5	0.0197 (11)	0.0105 (9)	0.0197 (11)	−0.0009 (9)	−0.0040 (9)	0.0007 (8)
C6	0.0149 (10)	0.0121 (9)	0.0162 (10)	−0.0015 (8)	0.0004 (8)	−0.0014 (7)
C7	0.0157 (10)	0.0137 (9)	0.0142 (10)	0.0024 (8)	−0.0008 (8)	−0.0014 (7)
C8	0.0135 (10)	0.0150 (10)	0.0180 (10)	−0.0025 (8)	−0.0008 (8)	−0.0027 (8)
C9	0.0127 (10)	0.0135 (9)	0.0192 (10)	−0.0006 (8)	0.0023 (8)	0.0003 (8)
C10	0.0127 (10)	0.0085 (9)	0.0151 (10)	0.0031 (8)	0.0003 (8)	−0.0006 (7)
C11	0.0118 (10)	0.0136 (9)	0.0193 (10)	−0.0027 (8)	−0.0012 (8)	0.0004 (8)
C12	0.0150 (10)	0.0138 (9)	0.0173 (10)	−0.0010 (8)	0.0023 (8)	0.0018 (8)
C13	0.0139 (10)	0.0163 (10)	0.0219 (11)	−0.0002 (9)	−0.0005 (8)	−0.0016 (8)
C14	0.0211 (11)	0.0196 (11)	0.0177 (10)	0.0066 (9)	−0.0016 (9)	−0.0022 (8)
C15	0.0250 (13)	0.0211 (11)	0.0179 (11)	0.0031 (10)	0.0048 (9)	0.0015 (9)
C16	0.0161 (10)	0.0176 (10)	0.0217 (11)	−0.0032 (9)	0.0035 (9)	0.0024 (8)

### Geometric parameters (Å, º)

**Table:**

Fe1—C1	2.032 (2)	C3—H3A	1.0000
Fe1—C1^i^	2.033 (2)	C4—C5	1.428 (3)
Fe1—C5	2.035 (2)	C4—H4A	1.0000
Fe1—C5^i^	2.035 (2)	C5—H5A	1.0000
Fe1—C3^i^	2.050 (2)	C7—C8	1.376 (3)
Fe1—C3	2.050 (2)	C7—C12	1.386 (3)
Fe1—C2	2.051 (2)	C8—C9	1.392 (3)
Fe1—C2^i^	2.051 (2)	C8—H8A	0.9500
Fe1—C4^i^	2.055 (2)	C9—C10	1.392 (3)
Fe1—C4	2.055 (2)	C9—H9A	0.9500
O1—C6	1.201 (3)	C10—C11	1.398 (3)
O2—C6	1.366 (2)	C11—C12	1.387 (3)
O2—C7	1.407 (2)	C11—H11A	0.9500
N1—C13	1.382 (3)	C12—H12A	0.9500
N1—C16	1.386 (3)	C13—C14	1.364 (3)
N1—C10	1.422 (3)	C13—H13A	0.9500
C1—C5	1.427 (3)	C14—C15	1.418 (3)
C1—C2	1.434 (3)	C14—H14A	0.9500
C1—C6	1.473 (3)	C15—C16	1.364 (3)
C2—C3	1.424 (3)	C15—H15A	0.9500
C2—H2A	1.0000	C16—H16A	0.9500
C3—C4	1.423 (3)		
			
C1—Fe1—C1^i^	180.0	C3—C2—Fe1	69.63 (13)
C1—Fe1—C5	41.09 (8)	C1—C2—Fe1	68.75 (12)
C1^i^—Fe1—C5	138.91 (8)	C3—C2—H2A	126.3
C1—Fe1—C5^i^	138.91 (8)	C1—C2—H2A	126.3
C1^i^—Fe1—C5^i^	41.09 (8)	Fe1—C2—H2A	126.3
C5—Fe1—C5^i^	180.0	C4—C3—C2	108.66 (19)
C1—Fe1—C3^i^	111.35 (8)	C4—C3—Fe1	69.90 (12)
C1^i^—Fe1—C3^i^	68.66 (8)	C2—C3—Fe1	69.74 (12)
C5—Fe1—C3^i^	111.27 (9)	C4—C3—H3A	125.7
C5^i^—Fe1—C3^i^	68.73 (9)	C2—C3—H3A	125.7
C1—Fe1—C3	68.65 (8)	Fe1—C3—H3A	125.7
C1^i^—Fe1—C3	111.34 (8)	C3—C4—C5	107.97 (18)
C5—Fe1—C3	68.73 (9)	C3—C4—Fe1	69.54 (12)
C5^i^—Fe1—C3	111.27 (9)	C5—C4—Fe1	68.84 (11)
C3^i^—Fe1—C3	180.0	C3—C4—H4A	126.0
C1—Fe1—C2	41.11 (8)	C5—C4—H4A	126.0
C1^i^—Fe1—C2	138.89 (8)	Fe1—C4—H4A	126.0
C5—Fe1—C2	69.15 (9)	C1—C5—C4	107.75 (18)
C5^i^—Fe1—C2	110.85 (9)	C1—C5—Fe1	69.36 (11)
C3^i^—Fe1—C2	139.37 (9)	C4—C5—Fe1	70.30 (11)
C3—Fe1—C2	40.63 (9)	C1—C5—H5A	126.1
C1—Fe1—C2^i^	138.89 (8)	C4—C5—H5A	126.1
C1^i^—Fe1—C2^i^	41.11 (8)	Fe1—C5—H5A	126.1
C5—Fe1—C2^i^	110.85 (9)	O1—C6—O2	123.84 (18)
C5^i^—Fe1—C2^i^	69.15 (9)	O1—C6—C1	126.17 (19)
C3^i^—Fe1—C2^i^	40.63 (9)	O2—C6—C1	109.97 (18)
C3—Fe1—C2^i^	139.37 (9)	C8—C7—C12	120.85 (19)
C2—Fe1—C2^i^	180.0	C8—C7—O2	116.66 (18)
C1—Fe1—C4^i^	111.29 (8)	C12—C7—O2	122.16 (18)
C1^i^—Fe1—C4^i^	68.71 (8)	C7—C8—C9	119.63 (19)
C5—Fe1—C4^i^	139.13 (9)	C7—C8—H8A	120.2
C5^i^—Fe1—C4^i^	40.87 (9)	C9—C8—H8A	120.2
C3^i^—Fe1—C4^i^	40.56 (9)	C10—C9—C8	120.67 (19)
C3—Fe1—C4^i^	139.44 (9)	C10—C9—H9A	119.7
C2—Fe1—C4^i^	111.44 (9)	C8—C9—H9A	119.7
C2^i^—Fe1—C4^i^	68.56 (9)	C9—C10—C11	118.55 (18)
C1—Fe1—C4	68.71 (8)	C9—C10—N1	120.40 (18)
C1^i^—Fe1—C4	111.29 (8)	C11—C10—N1	121.05 (17)
C5—Fe1—C4	40.87 (9)	C12—C11—C10	120.90 (19)
C5^i^—Fe1—C4	139.13 (9)	C12—C11—H11A	119.5
C3^i^—Fe1—C4	139.44 (9)	C10—C11—H11A	119.5
C3—Fe1—C4	40.56 (9)	C7—C12—C11	119.31 (19)
C2—Fe1—C4	68.56 (9)	C7—C12—H12A	120.3
C2^i^—Fe1—C4	111.44 (9)	C11—C12—H12A	120.3
C4^i^—Fe1—C4	180.00 (11)	C14—C13—N1	108.78 (19)
C6—O2—C7	119.52 (16)	C14—C13—H13A	125.6
C13—N1—C16	107.58 (17)	N1—C13—H13A	125.6
C13—N1—C10	126.34 (17)	C13—C14—C15	107.54 (19)
C16—N1—C10	126.08 (18)	C13—C14—H14A	126.2
C5—C1—C2	108.28 (18)	C15—C14—H14A	126.2
C5—C1—C6	127.65 (19)	C16—C15—C14	107.2 (2)
C2—C1—C6	123.89 (19)	C16—C15—H15A	126.4
C5—C1—Fe1	69.56 (12)	C14—C15—H15A	126.4
C2—C1—Fe1	70.14 (12)	C15—C16—N1	108.9 (2)
C6—C1—Fe1	122.25 (14)	C15—C16—H16A	125.6
C3—C2—C1	107.3 (2)	N1—C16—H16A	125.6

Symmetry code: (i) −*x*+1, −*y*+1, −*z*+1.
